# Discrimination of Carcinogens by Hepatic Transcript Profiling in Rats Following 28-day Administration

**DOI:** 10.4137/cin.s3229

**Published:** 2009-11-13

**Authors:** Hiroshi Matsumoto, Yoshikuni Yakabe, Koichi Saito, Kayo Sumida, Masaru Sekijima, Koji Nakayama, Hideki Miyaura, Fumiyo Saito, Masanori Otsuka, Tomoyuki Shirai

**Affiliations:** 1 Chemical Assessment Center, Chemicals Evaluation and Research Institute, Japan (CERI), 1600, Shimo-Takano, Sugito-machi, Kitakatsushika-gun, Saitama 345-0043, Japan; 2 Sumitomo Chemical Co., Ltd., Osaka, Japan; 3 Mitsubishi Chemical Safety Institute Ltd., Ibaraki, Japan; 4 Graduate School of Medical Sciences, Nagoya City University, Nagoya, Japan. Email: matsumoto-hiroshi@ceri.jp

**Keywords:** toxicogenomics, carcinogenicity, hepatocarcinogen, mutagenicity, microarray, cluster analysis

## Abstract

This study aimed at discriminating carcinogens on the basis of hepatic transcript profiling in the rats administrated with a variety of carcinogens and non-carcinogens. We conducted 28-day toxicity tests in male F344 rats with 47 carcinogens and 26 non-carcinogens, and then investigated periodically the hepatic gene expression profiles using custom microarrays. By hierarchical cluster analysis based on significantly altered genes, carcinogens were clustered into three major groups (Group 1 to 3). The formation of these groups was not affected by the gene sets used as well as the administration period, indicating that the grouping of carcinogens was universal independent of the conditions of both statistical analysis and toxicity testing. Seventeen carcinogens belonging to Group 1 were composed of mainly rat hepatocarcinogens, most of them being mutagenic ones. Group 2 was formed by three subgroups, which were composed of 23 carcinogens exhibiting distinct properties in terms of genotoxicity and target tissues, namely nonmutagenic hepatocarcinogens, and mutagenic and nonmutagenic carcinogens both of which are targeted to other tissues. Group 3 contained 6 carcinogens including 4 estrogenic substances, implying the group of estrogenic carcinogens. Gene network analyses revealed that the significantly altered genes in Group 1 included Bax, Tnfrsf6, Btg2, Mgmt and Abcb1b, suggesting that p53-mediated signaling pathway involved in early pathologic alterations associated with preceding mutagenic carcinogenesis. Thus, the common transcriptional signatures for each group might reflect the early molecular events of carcinogenesis and hence would enable us to identify the biomarker genes, and then to develop a new assay for carcinogenesis prediction.

## Introduction

A two-year carcinogenicity study with rodents is the standard method to assess the carcinogenic potential of chemicals. However, this model bioassay is time-consuming and extremely expensive to conduct. Therefore, development of a low-cost and short-term prediction test has been strongly desired. Successful models of such prediction methods are the medium-term liver bioassay established by Ito’s group^1^ and the carcinogenicity tests using transgenic or knock-out animals and others.^2^,^3^

Genotoxicity, which is one of important actions in the complex process of carcinogenesis, has been used as a useful marker to screen carcinogenicity of chemicals. However, the results of genotoxicity tests such as the Ames bacterial mutation assay do not always correlate with rodent carcinogenicities. Particularly in non-genotoxic carcinogens, the test results have shown significant differences in concordance with the rodent carcinogenicity results.^4^,^5^ Therefore, there has been a clear need to develop a novel assay for discriminating between potential carcinogens and non-carcinogens regardless of its genotoxicity.

Toxicogenomics has been expected to be a promising tool for investigation of toxicological properties of chemicals, because alteration of gene expression is the first event occurred in response to chemical exposure and lead to phenotypic changes. As for carcinogenicity, this tool enables us to obtain such properties of chemicals more precisely and precociously than the existing prediction methods. There have been previous reports on the prediction of potential carcinogenicity of chemicals in a short term toxicity study.^6^,^7^ For example, Nie et al reported that the carcinogenicity of nongenotoxic carcinogens can be predicted with 88.5% precision based on the expression data of 6 genes in male rats treated for 24 hours.^6^ Ellinger-Ziegelbauer et al also developed a prediction method of carcinogens with up to 88% accuracy using toxicogenomics analysis of short-term in vivo studies.^7^ However, those prediction methods were specialized in non-genotoxic carcinogen^6^ or developed within a small set of compounds.^7^

In general, it is possible that the gene expression profiles induced by chemical administration vary considerably among the mode of action of chemicals as well as the experimental conditions such as the dosage and duration. Since a number of mechanisms have been proposed for chemical carcinogenesis,^8^ it is presumed that carcinogenic chemicals are divided into more than one group based on the gene expression profiles relevant to their respective mechanism. Therefore, it is essential to use a suitable experimental procedure to find characteristic gene profiles for each group of carcinogens, regardless of toxicity test conditions.

The final goal of this study is to develop a short term, highly accurate and wide applicable prediction method of chemical carcinogenicity based on the hepatic gene expression profiles of rats treated with chemicals. Therefore, we conducted a research project in order to establish the optimized test protocol for toxicogenomics study,^9^,^10^ and then develop a database of the gene expression profiles of 85 carcinogens or non-carcinogens collected using the optimized protocol. In the present paper, as a part of the development of a prediction method for chemical carcinogenicity, we statistically analyzed the hepatic gene expression data of the rats in a large scale toxicity studies with 73 chemicals, and found to be classified in three major groups based on the information about genotoxicity and target tissues.

## Methods

### Test chemicals

At the beginning of our project, 85 test chemicals were selected by considering their chemical and toxicological diversity in order to find the universal gene expression profile for carcinogens from the US National Toxicology Program (NTP) database (http://ntp.mehs.nih.gov/) and the Chemical Carcinogenesis Research Information System (CCRIS) database (http://toxnet.nlm.nih.gov/cgi-bin/sis/htmlgen?CCRIS). In this study, 73 test chemicals were used (Table 1). The remaining 12 chemicals (tannic acid, quercetin, 2-nitro-p-phenyl-enediamine, 4-aminoazobenzene, DDT, 1-nitropyrene, dieldrin, 1-nitropropane, acetaminophen, 4-acetylami-nofluorene, carbon tetrachloride and glutaraldehyde) were reserved for the validation of the developed prediction method in further study. The results of the validation study will be reported elsewhere (Matsumoto et al in preparation).

Seventy-three test chemicals are composed by 21 mutagenic carcinogens (test No. C01 to C21 in Table 1), 26 non-mutagenic carcinogens (C22 to C47), 12 mutagenic non-carcinogens (NC01 to NCI2), 12 non-mutagenic non-carcinogens (NCI3 to NC24) and others (two non-carcinogens; NC25 and NC26, with unidentified mutagenicity). Eleven of 21 mutagenic carcinogens and 12 of 26 non-mutagenic carcinogens are hepatocarcinogens. Seventy-three test chemicals include six nitroso compounds (C01 to C06), two nitro compounds (C07 and C08), ten chlorinated organic compounds (CSV to C46), two heterocyclic amines (C12 and C13), two azocompounds (C09 and C10) and four poly cyclic aromatic hydrocarbons (C16 to C19), all of which have chemical structure relevant to carcinogenesis, and three peroxisome proliferators (C22 to C24), nine cytotoxic compounds (C14 and C15, C30 to C36), two steroidal estrogens (C27 and C28), two enzyme inducers (C25 and C26), all of which induce the known biological process relevant to carcinogens. The purity of test chemicals was >95%.

### Animals and treatment

Five-weeks-old male Fischer 344 (F344) rats were obtained from Charles River Laboratories Japan, Inc. (Atsugi, Japan) and allowed to acclimatise for 7 days prior to use. Rats were kept with pelleted chow food (CRF-1, Oriental Yeast, Co., Ltd) and tap water, ad libitum. Rats were randomly assigned to the two groups (treatment and control groups), and were administrated with each test chemical dissolved in the suitable vehicle by oral gavages once a day. Each of the groups was composed of 4 rats. The concurrent vehicle control groups were also prepared and treated as in the same manner with the test groups. The administration dose for each chemical was set around their minimum carcinogenic doses for carcinogens or maximum tolerable doses based on the information in NTP and CCRIS database as well as other literatures for non-carcinogens. As for 18 substances selected from carcinogens and non-carcinogens, they were administrated at four dosages to examine the dose dependency on the expression of the characteristic genes (Table 1). The maximum dose was set at half of LD50 and the remaining three doses were set up in a geometric ratio of 5. Rats were humanly sacrificed with CO_2_-O2 (4:1) gas inhalation at 3, 14 or 28 days after administration. The livers were immediately excised and weighed. Then, the liver samples from the left lateral lobe were sliced and immediately placed into RNAlater® (Ambion, Austin, TX, USA) for RNA extraction and thereafter gene expression analysis. A portion of the left lateral lobe was also taken for histopathology. Tissues were fixed in 10% neutral-buffered formalin, and subsequently sectioned and stained with hematoxylin and eosin.

### Oligo microarray

An oligo microarray, NEDO-ToxArray III, consisted of 6,709 unique genes and control genes was used for this study. The details of this custom array have been described previously^11^ and can also be seen on the project web site (http://www.nedo.go.jp/english/activities/2_sinenergy/l/p01004e.html).

### Gene expression data analysis

Gene expression data analysis was performed according to our previous study^11^ with a slight modification. In the present study, the cut-off value of each microarray data was determined by the mean signal intensity plus 2 standard deviations of three negative control probes (jojG, bioD and cre), and then the lowess normalization was done by the Statistical Microarray Analysis package of the R (http://www.r-project.org/).^12^,^13^ The mean signal intensities were calculated for four or three replicates (if the treated animal died) at each time point, and then subjected to characteristic gene selection.

In order to determine the signal log_2_ ratio with statistically significant difference in the gene expression level between the control and treatment groups, the variations of the signal intensity for four or three replicates at each time point were examined using the microarray data on 28-day treated rats with all samples. In more details, the coefficients of variation (CV) of the normalized signal intensity were calculated for all genes in the vehicle controls, which of the signal intensity were above the cut-off value for each micro-array data. Selection criteria of significantly changed genes were determined by power analysis.^14^ Power analysis was calculated using the mean CV of all samples by the following formula (1). Effective gene sets were selected the genes which were significantly changed in more than two carcinogens.

(1) $$1-\beta =P\left(Z\geq 1.96-\frac{\sqrt{n}\times \Delta x}{c}\right)$$

1-*β*: power, *Z*: criterion variable, *n*: sample size, Δ*x*: difference of signal ratio, *c*: coefficient of variation, 1.96 is rejection region at level of significance 5%.

### Cluster analysis for grouping of carcinogens

In order to divide carcinogens into groups, hierarchical cluster analysis was applied to the following selected gene data set. At first, differentially expressed genes in rat livers after treatment of between carcinogens and non-carcinogens were selected on the basis of Welch’s t-value^15^ as follows; gene data sets were selected from the significantly altered genes at each time point in the toxicity study using four different Welch’s t-values. Welch’s t-values were determined within a range of 2.0 to 3.3 at each time point so that the number of significantly changed genes was selected from 10 to 100. Then, reliability of the selected gene sets was confirmed by estimating the false discovery rate.^16^ Four gene data sets at each time point (total 12 gene data sets) were used in the analysis to examine the consistency of clustering of carcinogens. Using the gene data sets, hierarchical cluster analysis was performed for discriminating among different classes of carcinogens by Gene Maths (Applied Maths, Saint-Martens-Latem, Belgium). Classification of the chemicals was based on the shapes of the vicinity of the root of the dendrograms, and test chemical was defined as a carcinogen if it was clustered in the same cluster with other carcinogens in more than half of four cluster analyses with varied gene sets and was determined for each of 3rd, 14th and 28th day-analyses. Carcinogen groups were defined as the similar cluster belonging carcinogens in more than two analyses among 4 different gene sets at each of 3rd, 14th and 28th day-analyses. Hierarchical cluster analysis revealed two groups of carcinogens with clearly distinct gene expression profiles and a mixture group of several carcinogens and non-carcinogens, regardless of the administration period. Therefore, further cluster analysis was performed using these carcinogens and all non-carcinogens. Classification of the chemicals was also confirmed using the self-organizing map (SOM) method by the som package of the R and the principal component analysis (PCA) method by Gene Maths that were one of the unsupervised classification methods.

### Selection and functional analysis of the characteristic genes in each carcinogen group

The characteristic genes in each carcinogen group (Group 1 to 3) were selected by 3.5 of Welch’s t-value between of the carcinogens in each group and 27 non-carcinogens at each administration period. Then, functional analysis of the characteristic genes in each group was performed with IPA (http://www.ingenuity.com/), a web-delivered software for discovering, visualizing, and exploring relevant functions, pathways and networks. The characteristic genes at 28-day analyses were uploaded into IPA. LocusID of each gene was mapped to its corresponding gene object in the IPA Knowledge Base. Top 10 genes based on Welch’s t-values in each group were eligible for IPA analysis. These genes were then used as the starting point for generating biological functions, pathways and networks. Biological functions were calculated and assigned to different networks.

## Results

### In vivo toxicity studies

**Figure 1:**
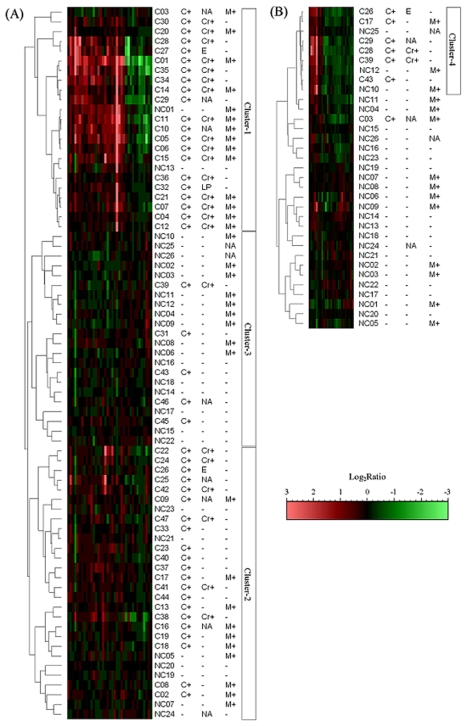
**A**) Result of hierarchical clustering using carcinogens (47) and non-carcinogens (26) in day 28. **B**) Result of hierarchical clustering using remaining carcinogens (7) and non-carcinogens (26) in day 28.

Male F344 rats were dosed daily via gavage for up to 28 days with 73 test chemicals including 23 hepatocarcinogens doses of which were documented to induce liver tumors in rats. In order to assess for pathological lesions induced during this subacute study, the livers were examined histopathologically. We found that 15 out of 23 hepatocarcinogens induced histological abnormalities such as modest hypertrophy and nuclear enlargement at 28 day post-treatment (Table 1). For example, dimethylnitrosamine treatment resulted in increasing necrosis in addition to the above findings. Also, cell infiltration was observed in some centrilobular areas, which was likely responsible for the weak reactive inflammation. In general, the modest histopathological lesions observed at the doses tested correspond to observations expected for genotoxic carcinogens. Together with the literature backed doses used, these pathological findings support the assumption that the animals would develop hepatic tumors if continued for further 20–24 months administration. However, the dose (0.5 mg/kg/day) of MMNG (COS) might not be adequate to identify a characteristic gene profile induced by the administration, because the number of genes significantly altered was extremely fewer than those of other carcinogens. This may be one of the factors that MNNG was not clustered with other test carcinogens (Fig. 1).

### Microarray data analysis

Following the background subtraction, a total of 5,664 to 6,683 genes were available for analysis on each of the microarray data at 28-day post-treatment. Overall, 5,292 genes were selected as common effective ones in all samples, and the mean CV values < 10%, 10%–30%, 30%–50%, ≥50% in 17%, 61%, 16% and 6% of the genes for the samples with four replicates (69 samples), respectively. Similarly, the mean CV values <10%, 10%–30%, 30%–50%, ≥50% in 30%, 62%, 6% and 2% of the genes for triplicates samples (clofibrate, d-mannitol, lithocholic acid and penta-chloroethane), respectively. As a result, the mean CV values of almost effective genes (94%) were <50% for the samples with four replicates, and those of 92% of the effective genes were <30% for ones with triplicates. From these results, the maximum values of potential variances of the signal intensity were estimated at 50% (n = 4) and 30% (n = 3), and then the power analysis was performed as follows: As for the four replicates samples, when Δx was 0.8 or more, power (1-β) was shown to be 80% or more which was statistically significant. Therefore, signal log_2_ ratio with a statistically significant difference was determined at ±0.8 (i.e. 1.8 or 1/1.8 by signal ratio). In case of the three replicates samples, 90% or more power was obtained by applying the Δx = 0.8. Therefore, it was considered that 0.8 of the signal log_2_ ratio was statistically significant in both three and four replicates samples. The selection criteria of the genes were determined as log_2_ ratio ≥ 0.8 or ≤ −0.8 to select robust changed genes in this study.

### Grouping of carcinogens

The primary gene sets at 3-, 14- and 28-day analyses were 392, 523 and 1,359, respectively, which were selected from significantly altered genes in at least two of 47 carcinogens in each of 3-, 14- and 28-day data. Then, the number of the genes was reduced using >2 of Welch’s t-value. With increasing Welch’s t-value, the differentially expressed genes between carcinogens and noncarcinogens were fewer and more statistically significant (Table 2A). For example, when Welch’s t-values of 2.3,2.5,2.8, and 3.0 were applied, 169, 116, 37, and 18 genes were selected for 28-day data. False discovery rate of each gene was estimated to be less than 0.05 in all gene sets. Therefore, cluster analysis was performed using all gene sets.

Figure 1(A) shows the hierarchical clustering of 73 chemicals against the expression data set of 37 genes selected with Welch’s t-value of 3.0 at 28-day analyses. Three clusters were formed as follows: The first cluster (cluster-1) was composed of 21 carcinogens and 2 non-carcinogens (NC01 and NCI3). Especially, many hepatocarcinogens were included in this cluster. The second (cluster-2) was also formed both by 21 carcinogens and 7 non-carcinogens. This group contains both non-mutagenic hepatocarcinogens and carcinogens which target tissues are except for rat liver. The third (cluster-3) showed a mixture of 17 non-carcinogens and 5 carcinogens. In order to test the consistency of clusters, cluster analysis was performed on relevant subsets of the characteristic genes selected by different t-values. The similar results were obtained with the other gene sets of 165, 116, and 33 genes (data not shown). Collectively, 20 and 22 carcinogens in cluster-1 and cluster-2 were categorized into the same cluster group in more than three times among four cluster analyses with different gene sets of 168 to 18 genes. As for the remaining 5 carcinogens, however, they were not classified into an independent cluster in all gene sets, and were always clustered with many non-carcinogens. With regards to 3- and 14-day data, similar results were observed in cluster analyses (data not shown). Consequently, 17 (36%) and 23 (49%) of 47 carcinogens belonged to the first and second cluster carcinogens in more than two analyses among 3rd, 14th and 28th day-analyses and referred to Group 1 and Group 2 in this paper, respectively (Table 1). Twelve of 17 carcinogens in Group 1 and 19 of 23 in Group 2 were selected in all of 3rd and 14th day analyses. The remaining 7 of 47 carcinogens did not form a consistent cluster. For example, ethinylestradiol (C28) and diethylstilbestrol (C27) were clustered into the first cluster in 28-day analysis, but not in 3- and 14-day analyses.

In order to examine whether these 7 carcinogens have the common gene expression profile or not, the gene set was newly selected by Welch’s t-value between 7 carcinogens and 26 non-carcinogens (Table 2B), and then hierarchical cluster analysis was retried. Figure 1(B) shows the hierarchical cluster analysis with 25 genes selected by Welch’s t-value of 2.5 between the 7 carcinogens and 26 non-carcinogens on 28-day data. The dendrogram was more complex compared to that in Figure 1(A), but 6 carcinogens except for MNNG (C03) belonged to the same cluster (cluster-4) with 3 non-carcinogens (NC09,11 and 25). As the same results were obtained form all of 3-, 14-and 28-day analyses, these five carcinogens were referred to Group 3 in this paper. As for MNNG, it did not make cluster with Group 3 carcinogens. Further validation was pursued using methods of PCA and SOM, and the similar clustering of carcinogens was observed by these methods (data not shown).

### Characteristic genes in the three carcinogen groups

**Figure 2:**
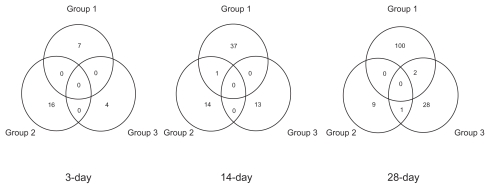
Overlap among the characteristic genes of three groups. Characteristic genes were selected by Welch’s t-value of 3.5.

The number of the characteristic genes in Group 1, 2 and 3, were 7, 16 and 4 in 3-day analysis, 38, 15 and 13 in 14-day analysis, and 102, 10 and 31 in 28-day analysis, respectively, when they were selected by 3.5 of Welch’s t-value. These genes did not overlap each other among three groups in 3- and 14-day analyses, and only one gene overlapped between Group 1 and 3 and between Group 2 and 3 in 28-day analysis (Fig. 2).

**Figure 3:**
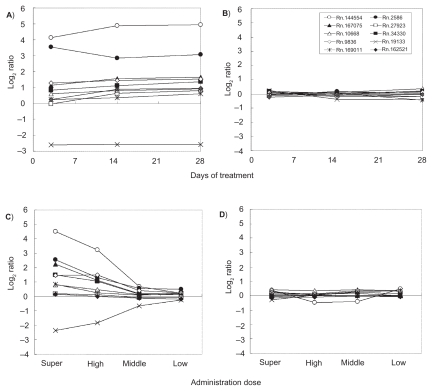
Gene expression profiles of top 10 characteristic genes which were selected in Group 1 during treatment period. **A**) thioacetamide (C35, group 1 carcinogen), and **B**) Lithocholic acid (NC19, non-carcinogen). **C**) Gene expression profiles of top 10 characteristic genes which were selected in Group 1 in the livers of male rats administered varying doses of thioacetamide (C35, group 1 carcinogen), and **D**) lithocholic acid (NC19, non-carcinogen).

Figure 3 (A) and (B) show time-course gene expression profiles of top 10 genes in Group 1 (Table 3) in male rat livers treated with thioacetamide (C35), and lithocholic acid (NC19). The up- and down-regulation of the characteristic genes was observed in nine and one out of them with treatment duration, and their expression changes reached a plateau at 3-day after administration of thioacetamide (Fig. 3A). On the other hand, such gene expression changes were not observed in the specimen treated with lithocholic acid. In thioacetamide treatment, the expression of the genes was also increased or decreased in a dose-dependent manner, but they were not changed dynamically by treatment with lithocholic acid (Fig. 3(C) and (D)). Similar findings were observed in other carcinogens in Group 1 carcinogens and non-carcinogens, though the fold change and time to reach a plateau varied in gene and dosage. The fold changes of top 10 genes in other groups were smaller than those of Group 1 carcinogens and the profiles were different from those of Group 1. As to Group 2 carcinogens, the expressions of eight and two in top 10 genes were increased and decreased with time, respectively (Table 4). The fold changes of them at 28-day post-administration were less and times to reach a plateau were faster compared to those of Group 1 carcinogens. As for Group 3 carcinogens, the expressions of four and six genes in top 10 genes were increased and decreased, respectively (Table 5). The fold changes of these genes at 28-day were comparable to those of Group 1 carcinogens, though the times to plateau were delayed and some genes did not reached to a plateau even after 28-day post-treatment. The expression of characteristic genes for Group 2 and 3 carcinogens was altered by administration of some other group carcinogens and non-carcinogens, but the expression pattern was different from the relevant group carcinogens.

**Figure 4:**
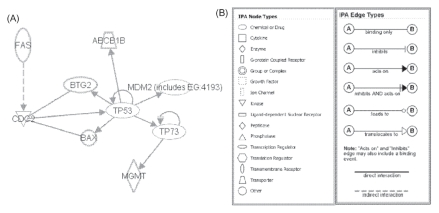
**A**) Connectivity map of the responses in the characteristic genes of carcinogens clustered to Group I by Ingenuity Pathway assistant analysis. **B**) explanation of the symbols, the edges, and their labels.

The top 10 characteristic genes in Group 1 had no a common point in their genes function (e.g. biological process in Gene Ontology) in spite of high score of Welch’s *t-*value compared with other two groups (Tables 4, 5, and 6). When biological networks of characteristic genes of Group 1 were analyzed using Ingenuity software, almost all the annotated genes except for Cyp2c40 were formed one network related with p53 gene which plays key role in carcinogenesis (Fig. 4(A)). As for Group 2, the characteristic genes did not connect with each other in network analysis. The top 10 characteristic genes of carcinogens clustered to Group 3 were formed a network via other non-characteristic genes such as NF-kB (Fig. 4(C)).

## Discussion

The present study showed that 47 carcinogens were clustered into three major groups by unsupervised hierarchical clustering. The first group was composed of 17 carcinogens, which were mainly genotoxic rat hepatocarcinogens. The second was formed by three subgroups, which were composed of 23 carcinogens exhibiting distinct properties in terms of genotoxicity and carcinogen-specific targeting, namely non-genotoxic rat hepatocarcinogens, and (non-)genotoxic carcinogens which the target were other tissues. The third consisted of 6 carcinogens including 4 estrogenic compounds (C16; benz[a]anthracene, C27; diethylstilbestrol, C28; ethinylestradiol, C40; 1,4-dichlorobenzene). The remaining one (MMNG, C03) was not clustered with other carcinogens. These clustering patterns were very consistent in analyzing with various gene data sets selected by different Welch’s t-values (2.3 to 3.0) or administration periods (3 to 28 days post-treatment). Furthermore, similar results were obtained using the other unsupervised classification analyses (i.e. SOM and PCA), confirming that these were neither accidental nor artificial by a specific statistical method, but were derived from common feature of the transcript profiling throughout the administration period. Overall, these results provide reproducible evidence for grouping of carcinogens based on the hepatic gene expression profiles of treated animals, suggesting that this classification was universal wide range of carcinogens with different chemical structure and biological process as well as administration period.

The test substances used in this study included a wide variety of carcinogens in terms of the target organ and biological activities relevant to carcinogenicity (e.g. mutagenicity). Therefore, we investigated the relationship between these features of the test chemicals and the grouping of them by unsupervised hierarchical clustering analysis. The hepatic gene expression profiles of the treated rat clearly discriminated the Group 1 from other groups. In this study, 11 of 47 test carcinogens are mutagenic rat hepatocarcinogens, and all of them were classified into the Group 1, indicating that this group was characterized by rat hepatocarcinogens with mutagenic activity. Although 6 carcinogens belonging to this group were Ames test negative, possible metabolites of acetamide (C30) and methyl carbamate (C34) have been reported to be mutagenic (IARC monographs vol71/mono71-59; http://monographs.iarc.fr.,),^17^ or verified positive responses in other mutagenicity assays have been obtained for methapyrilene HCl (C29), urethane (C36) and 1,4-dioxane (C32) from the NTP reports and the CCRIS database. In addition, as for some nonmutagenic chemicals, it is known that indirectly induced oxidative base modifications could contribute to induction of DNA mutations.^18^ Therefore, these chemicals or their metabolites might exert genotoxic activity to the liver of treated rats through an indirect mechanism. In this group, two non-carcinogens (NC01 and NC13) were also classified. Unsupervised hierarchical clustering analysis revealed that 4-(chloroacetyl)acetanilide (NC01) which contains a carboxamide functional group was located adjacent to 2-AAF (C11), which is also a carboxamide compound. Therefore, the structural similarity between them exerted analogous effects on the hepatic gene expression of the administrated rats. On the other hand, the reasons why 3-Chloro-p-toluidine (NC13) was classified into Group 1 are unknown. Overall, these indicate that Group 1 consists of mainly mutagenic hepatocarcinogens, suggesting that the gene expression profiles clearly delineate the early response to administration of mutagenic hepatocarcinogens.

Chemically induced rat liver cancer proceeds through multiple, distinct initiation-promotion-progression stages and mutation of the suppressor p53 gene has been found in relatively early preneo-plastic lesions in the rat liver.^19^ In the present study, gene network analysis using the characteristic genes in Group 1 revealed that almost all the annotated genes were formed one network related with p53 gene (Fig. 4(A)). The p53 network contained several key genes as follows: The Bax gene encodes the pro-apoptotic Bax protein that has p53-binding element in their promoter region which is capable of p53-dependent transcriptional activation.^20^ The Abcblb gene is reported that a membrane-associated protein encoded by this gene is a member of the superfamily of ATP-binding cassette (ABC) transporters, and this gene have p53-binding site spanning from base pairs −199 to −180 in the rat Abcblb promoter that is essential for basal and xenobiotics-inducible promoter activities.^21^ The Btg2 protein is a member of the BTG/Tob family which has structurally related proteins that appear to have antiproliferative properties and involved in the regulation of the G1/S transition of the cell cycle,^22^ and this gene is up-regulated by p53 protein.^23^ DNA repair gene, Mgmt, is regulated by p73-dependent transcriptional activation which related with p53 pathway.^24^ The Tnfrs6 (Fas) protein is a member of the TNF-receptor superfamily and contains a death domain. It has been shown to play a central role in the physiological regulation of programmed cell death, and has been implicated in the pathogenesis of various malignancies and diseases of the immune system (reviewed in).^25^ In this pathway, Tnfrs6 is interacted with Btg2, Bax, and p53 via Cdc2 (Fig. 4(A)). These results show that most of characteristic genes in the carcinogens belong to the Group 1 are formed network related with p53, suggesting that they induced various biological process such as cell cycle arrest, DNA repair, apoptosis, immune response, and drug metabolism, which are defensive responses to carcinogenesis. Histological abnormalities which were frequently observed in the livers of rat treated with Group 1 carcinogens support this hypothesis.

The Group 2 was divided into two large groups, one consisting of nonmutagenic (Group 2A), and the other of mutagenic ones (Group 2B). Additionally, the nonmutagenic carcinogens in the Group 2A were largely separated into two subgroups, rat hepatocarcinogens and others. The first subgroup (Group 2A-1) comprised three rat hepatocarcinogens (clofibrate; C22, di(2-ethylhexyl)phthalate; C24, alpha-hexachlorocyclohexane; C42), whereas the other (sub)group also contained several nonmutagenic hepatocarcinogens. The second (Group 2A-2) consisted of five nonmutagenic carcinogens (d-Limonene; C33, Di(2-ethylhexyl)adipate; C23, 1,4-Dichlorobenzene; C40, Aldrin; C37, Benzo[a]pyrene; C17, Trichloro-ethylene; C44), though several nonmutagenic carcinogens were classified in other clusters. In this study, unlike the mutagenic rat hepatocarcinogens, nonmutagenic ones were incorrectly classified in some clustering conditions, van Delft et al^26^ examined the gene expression patterns of HepG2 cells exposed to both genotoxic and non-genotoxic carcinogens. Then they reported that clustering analysis almost perfectly separated genotoxic carcinogens from nongenotoxic ones, but a few nongenotoxic carcinogens were placed in the wrong clusters (i.e. in between genotoxic carcinogens). It has been reported that modulation of gene expression profiles by nonmutagenic carcinogens are extremely complicated, because the modes of action of carcinogenesis are numerous and very diverse.^27^ Gene network analysis in the present study did not reveal the distinct network underlying early responses of rat livers induced by nonmutagenic carcinogens. Thus, the discrimination of nonmutagenic carcinogens based on the gene expression profiles could be feasible, but some chemicals might be ambiguously classified dependent on the analysis conditions.

Another subgroup (2B) was comprised of 5 mutagenic carcinogens (PhIP; C13, 3-Methylcholanthrene; C19, 7,12-Dimethylbenz [a]anthracene; C18, 4-Nitroquinoline-1-oxide; C08, N-Ethyl-N-nitrosourea; C02), which the target tissue was not livers. Regarding mutagenicity of the test chemicals, the Group 1 carcinogens were also Ames test positive. The separation between Group 2B and Group 1 could be explained by the poor induction of carcinogenesis-related gene expression in the non-target tissue (i.e. livers) by Group 2B chemicals, whereas Group 1 chemicals were all strong inducers of them (Fig. 1). Several reports showed that renal carcinogen treatment produced more significant alteration of gene expression in kidney (target tissue) than in liver (non-target tissue) of rats, suggesting that these differential alterations could be the underlying mechanisms for the tissue-specific carcinogenicity of chemicals.^28^,^29^ Therefore, the present study, which examined the hepatic gene expression changes of rats treated with carcinogens, could distinguish the early responses to administration of hepatocarcinogens as well as the other ones, although less profound alteration was observed in case that the livers were non-target tissues.

Six carcinogens in the last group (Group 3) were grouped in clusters not by the first analysis but the second one of hierarchical clustering. Therefore, the transcriptional similarities of significantly altered genes in the treated rats were lower than those of other groups. Yet, four out of these six chemicals (benz[a]anthracene; C16, diethylstilbestrol; C27, ethinylestradiol; C28, 1,4-dichlorobenzene; C40) have an estrogenic activity, implying the group of estrogenic carcinogens. The evidence is strong that estrogen-modulating effects are closely related to carcinogenicity or mutagenicity.^30^ With regards to the expression altered genes, significant down-regulation of steroid 5 alpha-reductase gene, which catalyzes the conversion of testosterone to dihydrotestosterone, was observed in the list of top 10 genes. Although the molecular mechanisms responsible for estrogen carcinogenicity are not well understood, this might partly show the molecular mechanisms underlying estrogen-associated carcinogenesis. Further work needs to be done to confirm this assumption.

This study provides proof-in-principle that carcinogens could be classified into three large groups based on the hepatic gene expression profiles of rats following the administration, based on their genotoxic properties or target tissues. In regulatory toxicology, this finding can be help to understand the mode of action for carcinogenicity of a wide variety of chemicals, especially nongenotoxic ones. Moreover, by applying to the current in vivo toxicity test using rats, this finding will pave the way for use of gene transcription biomarker signatures for screening of carcinogenic potential of test chemicals. Especially if a subset of genes which were selected in this study could be characterized as robust predictor genes, this may lead to the development of a new screening assay for screening chemicals on their carcinogenetic properties with higher predictability and broader applicability.

In short, 47 carcinogens were clustered into three major groups based on their mutagenicity and target tissues by unsupervised hierarchical clustering using the hepatic gene expression profiles of rats treated for 28 days. The common transcriptional signatures for each carcinogen group might reflect the early molecular events of carcinogenesis and hence would enable us to identify the biomarker genes for carcinogenic chemicals, and then to develop a new assay for carcinogenesis prediction.

## Figures and Tables

**Table 1:** Summary of the toxicity tests with 73 test chemicals, their carcinogenic properties, and carcinogen group clustered in this study.

Test No.	Name	Source*1	Toxicity test			Carcinogenic properties			Carcinogen group no.*8
			Dose*2	Vehicle*3	Histopath.*4	Car.*5	Hepatocar.*6	Muta.*7	
C01	Diethylnitrosamine	B	20(30,6, 1.2, 0.24)	DW	FICI, SCN	+	+	+	1
C02	*N*-Ethyl-*N*-nitrosourea	A	3	DW	NO	+	−	+	2
C03	MNNG	B	0.5	DW	NO	+	NA	+	other
C04	*N*-Nitrosodimethylamine	A	0.2	DW	NO	+	+	+	1
C05	*N*-Nitrosomorpholine	B	10 (141, 28.2, 5.64, 1.13)	DW	FICI, SCN	+	+	+	1
C06	*N*-Nitrosopiperidine	G	10	DW	DHH, FICI	+	+	+	1
C07	2-Nitropropane	A	40	CO	GI	+	+	+	1
C08	4-Nitroquinoline-1-oxide	A	2 (5, 1, 0.2, 0.04)	CO	NO	+	−	+	2
C09	4-Dimethylaminoazobenzene	C	50	CO	NO	+	NA	+	2
C10	3′-Methyl-4-dimethylamino azobenzene	B	50	CO	AH, PHH,	+	NA	+	1
C11	2-Acetylaminofluorene	A	6	CO	DHH, VH	+	+	+	1
C12	MelQx	H	20	1% CMC	NO	+	+	+	1
C13	PhIP	H	5	1% CMC	NO	+	−	+	2
C14	Furan	C	10	CO	AH, HHN VH	+	+	+	1
C15	Safrole	C	300	CO	ECH, SCN	+	+	+	2
C16	Benz[a]anthracene	B	50 (100, 20, 4, 0.8)	CO	NO	+	NA	+	3
C17	Benzo[a]pyrene	C	15	CO	NO	+	−	+	2
C18	7,12-Dimethylbenz[a] anthracene	A	1 (30, 6, 1.2, 0.24)	CO	NO	+	−	+	2
C19	3-Methylcholanthrene	A	2	CO	NO	+	−	+	2
C20	Quinoline	E	25(30,6, 1.2, 0.24)	CO	HHN, IMF, SCN	+	+	+	1
C21	2,4-Diaminotoluene	D	10	DW	PHH	+	+	+	1
C22	Clofibrate	A	250 (275, 55, 11, 2.2)	CO	PHH	+	+	−	2
C23	Di(2-ethylhexyl) adipate	C	1,000	CO	NO	+	−	−	2
C24	Di(2-ethylhexyl) phthalate	A	300	CO	PHH	+	+	−	2
C25	Phenobarbital	C	100 (110, 22, 4.4, 0.88)	DW	PHH	+	NA	−	3
C26	Phenytoin	B	160	DW	NO	+	E	−	2
C27	Diethylstilbestrol	B	10	CO	VH	+	E	−	3
C28	Ethinylestradiol	A	0.5	CO	ATM	+	+	−	3
C29	Methapyrilene HCl	A	50	DW	DHH, FICI, HHN, IMF, SCN	+	NA	−	1
C30	Acetamide	A	1,180	DW	NO	+	+	−	1
C31	Butylated hydroxyanisole	C	750 (1,000, 200, 40, 8)	CO	NO	+	+	−	2
C32	1,4-Dioxane	C	1,000 (1,000, 200, 40,8)	DW	PHH	+	LP	−	1
C33	d-Limonene	A	1,000 (800, 160, 32, 6.4)	CO	NO	+	−	−	2
C34	Methyl carbamate	B	500	DW	AH, GI, IMF	+	+	−	1
C35	Thioacetamide	C	20 (25, 5, 1, 0.2)	DW	AH, HHN	+	+	−	1
C36	Urethane	A	80	DW	NO	+	+	−	1
C37	Aldrin	I	0.3	CO	NO	+	−	−	2
C38	Chlorendic acid	B	100	DW	NO	+	+	−	2
C39	Chloroform	A	90	CO	NO	+	+	−	3
C40	1,4-Dichlorobenzene	B	300	CO	PHH	+	−	−	2
C41	Hexachlorobenzene	A	5	CO	NO	+	+	−	2
C42	alpha-Hexachloro	C	20	CO	PHH	+	+	−	2
C43	cyclohexane	B	200	CO	NO	+	−	−	3
C44	Pentachloroethane	A	700	CO	NO	+	−	−	2
C45	Trichloroethylene	A	100	CO	NO	+	−	−	2
C46	Tetrachloroethylene	A	300	DW	NO	+	NA	−	2
C47	Trichloroacetic acid D,L-Ethionine	C	30 (250, 50, 10,2)	CO	NO	+	+	−	2
NC01	4-(Chloroacetyl)acetanilide	A	250	CO	NO	−	−	+	−
NC02	2-Chloroetahnol	A	40	DW	NO	−	−	+	−
NC03	2-Chloromethylpyridine HCI	B	150	DW	NO	−	−	+	−
NC04	2-Chloro-*p*-phenylenediamine SO4	A	100	1% CMC	NO	−	−	+	−
NCO5	1-Chloro-2-propanol	F	100	DW	NO	−	−	+	−
NC06	2,6-Diaminotoluene	A	10	DW	NO	−	−	+	−
NC07	8-Hydroxyquinoline	B	25	CO	NO	−	−	+	−
NCO8	4-Nitroanthranilic acid	B	1,000	CO	GI	−	−	+	−
NC09	1-Nitre-naphthalene	B	100	CO	IMF	−	−	+	−
NC10	4-Nitro-*o*-phenylenediamine	A	250	1% CMC	NO	−	−	+	−
NC11	*p*-Phenylenediamine 2HCl	A	60	DW	NO	−	−	+	−
NC12	2,5-Toluenediamine SO4	B	50	1% CMC		−	−	+	−
NC13	3-Chloro-*p*-toluidine	B	300	CO	AH, PHH	−	−	−	−
NC14	L-Ascorbic acid	C	1,000 (1,000, 200, 40, 8)	DW	NO	−	−	−	−
NC15	Aspirin	A	27	CO	NO	−	−	−	−
NC16	Caprolactam	A	375	DW	NO	−	−	−	−
NC17	Indomethacin	C	5	CO	NO	−	−	−	−
NC18	Lindane	A	10	CO	NO	−	−	−	−
NC19	Lithocholic acid	C	1,000 (750, 150, 30, 6)	5% AGS	NO	−	−	−	−
NC20	D-Mannitol	C	1,000	DW	NO	−	−	−	−
NC21	DL-Menthol	D	1,000	CO	PHH	−	−	−	−
NC22	Phthalamide	B	1,000	CO	NO	−	−	−	−
NC23	Sodium benzoate	C	1,000	DW	GI	−	−	−	−
NC24	Alpha-Tocopherol	C	1,000 (1,000, 200, 40, 8)	CO	NO	−	NA	−	−
NC25	Benzoin	C	500	5% AGS	NO	−	−	NA	−
NC26	lodoform	C	200	CO	NO	−	−	NA	−

*1 A; Sigma-Aldrich Co. (St. Louis, MO, USA), B; Tokyo chemical Co., Ltd (Tokyo, Japan), C; Wako pure chemical Industries, Ltd. (Osaka, Japan), D; Junsei chemical Co., Ltd (Tokyo, Japan), E; Kishida Chemical Co., Ltd (Osaka, Japan), F; Fluka Chemical Co. (Buchs, Switzerland), G; Kanto chemical Co., Inc. (Tokyo, Japan), H; Nard institute Ltd. (Hyogo, Japan), I; AccuStandard Inc. (New Haven, CT, USA).

*2 mg/kg/day.

*3 5% AGS; 5.0 w/v % Arabic gum solution, CO; Corn oil, DW; Distilled water, 1% CMC; 1% Carboxymethylcellulose sodium solution.

*4 Histo-pathology: AH; Apoptosis of hepatocytes, ATH; Atrophy of hepatocytes, DHH; Diffuse hypertrophy of hepatocytes, ECH; Eosinophilic change of hepatocytes, FICI; Focal inflammatory cell infiltrates in liver, GI; glycogen increment, IMF; Increment of mitotic figure in hepatocytes, HHN; Hypertrophy of hepatocyte nuclear, NO; No histological abnormalities, PHH; Periportal hypertrophy of hepatocytes, SCN; Single cell necrosis of hepatocyes, VH; Vacuolization of hepatocytes.

*5 Carcino-genicity.

*5 E; Equivocal, NA; Not available data, LP; Limited positive.

*6 Hepato-carcinogenicity.

*7 Muta-genecity

*8 The results based on the present study.

**Table 2A-B:** Number of differentially expressed genes in rat liver after treatment between carcinogens and non-carcinogens.

A.			
t-value	Days after treatment		
	3	14	28
2.0	50	–	–
2.3	33	–	169
2.5	24	76	116
2.8	15	52	37
3.0	–	42	18
3.3	–	19	–
**B.**			
2.0	28	54	72
2.3	16	33	42
2.5	11	22	25
2.8	6	–	16

**Table 3:** Top 10 characteristic genes of group 1 carcinogens at 28th day.

Gene name	Unigene ID	Description	Gene ontology (Biological process)	Welch’s t-value	Mean of log_2_ ratio
ATP-binding cassette, sub-family B (MDRTAP), member 1B	Rn. 144554	member of the ATP-binding cassette (ABC) protein superfamily; may play a role in drug disposition	response to ionizing radiation, response to arsenic, drug transport	7.27	3.88
Cytochrome P450 2c13	Rn.2586	polymorphic cytochrome P450 isozyme with male specific expression	electron transport	7.25	1.27
Transcribed locus	Rn. 167075	–	–	5.27	1.44
B-cell translocation gene 2, anti-proliferative	Rn.27923	an anti-proliferative protein; interacts with Pick1 and may have a role in PKC-mediated extracellular signal transduction and cellular differentiation	protein amino acid methylation, negative regulation of apoptosis, neuron differentiation	5.26	0.44
Bcl2-associated X protein	Rn. 10668	Bcl2-related gene; involved in the regulation of apoptotic cell death	negative regulation of fibroblast proliferation, outer mitochondrial membrane organization and biogenesis, induction of apoptosis	5.26	0.72
Transcribed locus	Rn. 34330	–	–	5.23	0.88
O-6-methylguanine-DNA methyltransferase	Rn.9836	enzyme involved in DMA repair of O(6)-alkylguanine which is the major mutagenic and carcinogenic lesion in DNA	DNA ligation, DNA repair, regulation of caspase activity	5.18	1.50
Similar to indolethylamine N-methyltransferase	Rn. 19133	–	–	5.08	−1.59
Similar to Deoxyuridine 5-triphosphate nucleotidohydrolase	Rn. 169011	–	–	4.89	0.49
Tumor necrosis factor receptor superfamily, member 6	Rn. 162521	Tnfsf6/Fasl receptor	induction of apoptosis, apoptosis, activated T cell apoptosis	4.89	0.47

**Table 4:** Top 10 characteristic genes of group 2 carcinogens at 28th day.

Gene name	Unigene ID	Description	Gene ontology (Biological process)	Welch’s t-value	Mean of log_2_ ratio
Glutamyl-prolyl-tRNA synthetase	Rn.21240	–	prolyl-tRNA aminoacylation, protein complex assembly, tRNA aminoacylation for protein translation	4.28	0.74
CCAATenhancer binding protein (CEBP), beta	Rn.6479	transcription factor that binds to CCAATT motif on DNA and may facilitate IL-6 induced transcriptional activation	transcription from RNA polymerase II promoter, neuron differentiation, fat cell differentiation	4.16	0.23
CD63 antigen	Rn.11068	human homolog facilitates endocytosis of H,K-ATPase beta-subunit; may play a role in protein trafficking	positive regulation of endocytosis	3.87	−0.38
Lymphotoxin B receptor	Rn. 19329	–	lymph node development, signal transduction, positive regulation of l-kappaB kinase/NF-kappaB cascade	3.86	0.66
Similar to putative protein, with at least 6 transmembrane domains, of ancient origin (58.5 kD) (3N884) (predicted)	Rn. 152690	–	metabolism	3.80	0.15
Alpha-fetoprotein	Rn.9174	plasma protein expressed in the fetal liver and yolk sac	progesterone metabolism, sexual reproduction, ovulation (sensu Mammalia)	3.69	0.28
AFG3(ATPase family gene 3)-like 1 (yeast) (predicted)	Rn.41391	–	–	3.67	0.64
Transcribed locus	Rn. 120914	–	–	3.61	−0.25
Selenocysteine lyase	Rn. 23954	mouse homolog catalyzes the decomposition of L-selenocysteine to produce L-alanine and selenium; may function to deliver elemental selenium to selenophosphate synthetase for selenoprotein biosynthesis	metabolic process, amino acid metabolism, selenocysteine catabolism	3.54	0.54
Transcribed locus	Rn. 166039	–	–	3.51	0.50

**Table 5:** Top 10 characteristic genes of group 3 carcinogens at 28th day.

Gene name	Unigene ID	Description	Gene ontology (Biological process)	Welch’s t-value	Mean of log_2_ ratio
Transcribed locus	Rn. 164817	–	–	5.88	1.11
probasin	Rn.9862	displays androgen dependent expression in prostate epithelial cells	–	5.57	−1.68
cd36 antigen	Rn. 102418	fatty acid translocase; involved in long-chain fatty acid (LCFA) transport; important in fatty acid metabolism and insulin function	blood coagulation, fatty acid transport, long-chain fatty acid transport	4.98	1.97
Transcribed locus	Rn. 164639	–	–	4.60	−0.21
Glial cell line derived neurotrophic factor family receptor alpha 1	Rn. 88489	binds glial cell line-derived neurotrophic factor (GDNF) and mediates Ret protein-tyrosine kinase receptor phosphorylation and activation	transmembrane receptor protein tyrosine kinase signaling pathway, nervous system development, cell surface receptor linked signal transduction	4.57	−0.73
RT1 class II, locus Ba	Rn. 25717	may play a role in antigen presentation	antigen processing and presentation of peptide or polysaccharide antigen via MHC class II, immune response	4.46	−0.54
Dehydrogenasereductase (SDR family) member 7	Rn. 119024	exhibits oxidoreductase activity; involved in metabolic process (inferred)	metabolic process	4.46	−2.46
PYD and CARD domain containing	Rn.7817	may play a role in apoptosis	induction of apoptosis, regulation of caspase activity, positive regulation of interleukin-1 beta secretion	4.45	−0.45
Steroid 5 alpha-reductase 1	Rn.4620	catalyzes the conversion of testosterone to dihydrotestosterone; required for male sex differentiation	sex determination, androgen biosynthesis, male sex differentiation	4.43	1.07
Prolactin receptor	Rn.9757	high affinity receptor for prolactin (PRL); may mediate prolactin functions in brain including reproduction, sexual behavior, feeding behavior, and maternal behavior; may play a role in regulation of GnRH secretion, firing rate of hypothalamic neurons, metabolism of neurotransmitters and neuropeptides, oxytocin release, enzyme activities in neurons, and glial cell proliferation	embryo implantation, regulation of epithelial cell differentiation, regulation of cell adhesion	4.38	2.01
